# Upgrading Big Brother: Local Strategic Adaptation in China’s Security Industry

**DOI:** 10.1007/s12116-021-09342-9

**Published:** 2021-10-06

**Authors:** Jingyang Huang, Kellee S. Tsai

**Affiliations:** Division of Social Science, Hong Kong University of Science and Technology, Clear Water Bay, Kowloon, Hong Kong SAR

**Keywords:** China, Industrial upgrading, Local development, Surveillance, Technological innovation

## Abstract

What are the circumstances under which latecomer firms can develop indigenous innovative capacity and compete globally? China’s construction of a vast domestic security apparatus has contributed to it becoming the world’s largest supplier and consumer of video surveillance products and services. It has also produced several globally competitive companies, including those engaged in digital surveillance. Although security equipment producers initially emerged in Guangdong province, China’s leading technology companies are concentrated in Zhejiang province. This comparative study is motivated by the puzzle of why Guangdong, which has a larger security equipment industry with more local investment and earlier introduction of foreign technology, has lagged behind Zhejiang in technological upgrading. We trace this provincial variation to the *policy choices of local state bureaucracies* and the *business strategies of local enterprises*. While macro-level variables such as market demand and establishing national industry standards are important for industrial development, this study demonstrates the critical role of local technocrats and entrepreneurs in facilitating technological innovation in a rapidly evolving surveillance regime. Our analysis also reveals how latecomers to a technology-intensive industry were able to adapt their products strategically to meet the technical demands of a major segment of the domestic market, China’s public security sector.

China is a living example that totalitarian forms of surveillance appear to be entirely compatible with voracious capitalist economic development.

       - David Murakami Wood, “Globalization and Surveillance” (2012)

Distributional politics tend to slow innovation; security politics tend to accelerate innovation.

       - Mark Zachary Taylor, *Politics of Innovation *(2016)

## Introduction

It is well-established that strong states in late industrializing countries may leverage certain “advantages of backwardness” when they prioritize national economic growth and mobilize resources to develop strategic sectors (Gerschenkron 1962). Familiar examples include Weimar Germany, Meiji Japan, and the post-war East Asian capitalist economies. When it comes to technological innovation, however, traditional developmental state instruments have proven to be less effective than in earlier phases of catch-up industrialization (Wong 2011). This presents a challenge for newly industrialized countries seeking to climb the global value chain (GVC). While globalization has expanded the range of producers across territorial borders, multinational corporations (MNCs) with leading edge technology dominate the most lucrative and value-added segments of the production cycle (Gereffi et al. 2005; Whittaker et al. 2020). What are the circumstances under which latecomer firms can develop indigenous innovative capacity and compete globally? The recent rise of China’s video surveillance industry, combined with subnational variation in sectoral features, offers counterintuitive insights into the political economy of industrial upgrading.

In China’s political context, surveillance equipment represents a strategic sector. Since the late 1990s, the Chinese Communist Party (CCP) has emphasized the critical importance of maintaining domestic social stability (*weiwen*) and devoted considerable administrative and financial resources towards that goal. The construction of an extensive network of video surveillance units is one of the most tangible expressions of the party-state’s effort to monitor its citizens (Cai 2019; Xu 2021). This political imperative has had economic implications. With the installation of over 200 million “eyes in the sky,” China is the world’s largest market for video hardware and possesses the second highest density of security cameras after the United States.^1^ It has also become a global leader in the industry. By 2020, seven Chinese firms ranked among the top fifteen global security companies.^2^ The top two, Hikvision and Dahua, are among China’s leading technology enterprises. Both have introduced innovations in surveillance technology—including facial recognition, multitarget tracking, advanced 360-degree cameras, patented extended network switches for long-distance transmission, and cost-effective coding of images. Hikvision and Dahua represent key original brand manufacturers (OBMs) in the surveillance equipment GVC.^3^

The rapid rise of *globally competitive* video surveillance companies in China is a relatively recent phenomenon, however. Security equipment producers first emerged in Guangdong province during the 1990s. This is not surprising given the high concentration of electronics manufacturing in the Pearl River Delta. Coupled with access to international production networks, foreign technology, and the country’s largest share of in-bound foreign direct investment (FDI), Guangdong’s economic development has been “one step ahead” of the rest of the country since the earliest years of economic reform (Vogel 1989). Moreover, Guangdong possesses conditions that are conducive to technological innovation in the security equipment industry: it has the largest volume of video camera manufacturers, the greatest cluster of foreign security equipment technology companies, and, as a result, vibrant intrasectoral competition. Yet even after several decades of development, none of the dominant international companies are indigenous to Guangdong. Instead, China’s top globally ranked security technology companies were founded in Hangzhou city in Zhejiang province.^4^ This paper seeks to explain how China was able to produce cutting-edge security companies within a relatively short time frame, and why Zhejiang, rather than Guangdong, came to lead this process.

In the study of comparative capitalism, contemporary China is typically characterized as “state capitalist,” meaning that while most of the economy is open to market competition, the state retains ownership over enterprises in strategic areas, exercises political control over “national champions,” and promotes select sectors through industrial policies (Naughton and Tsai 2015). The mechanisms underlying rapid technological upgrading in the surveillance sector deviate from this macro-level depiction of China’s political economy, however. Rather than consequentialist intervention by the state to promote technological innovation, we contend that the rapid industrial upgrading of security equipment companies is a positive externality of the Public Security Bureau (PSB)’s mandate to maintain social stability, coupled with strategic adaptation by latecomer firms to market opportunities. Unlike familiar developmental state explanations of industrial growth, in the case of China’s public surveillance sector, front-line PSB technocrats rather than industrial planners were authorized with sourcing the highest quality equipment. The state’s overarching priority of augmenting its surveillance capacity drove its procurement of technologically advanced security products during the initial phase of public surveillance construction. For this reason, foreign companies dominated China’s security equipment market during the 1990s. Over time, however, domestic firms became more attuned to local information about what it would take to develop products sufficiently sophisticated to secure large government contracts, and some were operating in a local policy context that enabled them to adapt the technical standards of their products accordingly.

Our argument rests on three main observations. First, China’s *public security apparatus* has become the principal buyer-driven force for security equipment and technical services, setting quality standards for both domestic and foreign companies. Second, *professional technocrats* in the Public Security Bureau (rather than industrial policy bureaucrats) represent the key actors in promoting technological innovation in surveillance products. Third, we contend that *strategic adaptation* by both firms and local officials has yielded variation in industrial upgrading at the subnational level. Relatedly, the *timing of industrial development* is an important contextual variable such that sectoral market conditions at the outset mediate the subsequent strategic choices of government officials and local entrepreneurs.

In brief, the strategic adaptation process in Guangdong led local companies to adopt a *commercial market–oriented* business strategy before the 2000s. With a first-mover advantage, local workshops learned how to re-engineer foreign products and reproduced them at a lower cost. Guangdong’s early manufacturers thus secured a large market share. After the mid-2000s, however, this same strategy limited the competitiveness of Guangdong’s products in national public security projects, and the emergence of a more restrictive regulatory environment disincentivized the introduction of new products. By contrast, Zhejiang’s pattern of strategic adaption led Hangzhou’s companies to adopt a business strategy oriented towards serving as *subcontractors for publicly funded initiatives*. Rather than competing for a share of the civilian market already dominated by Guangdong’s manufacturers, Zhejiang’s newer and loosely regulated security companies opted to meet the technical requirements of public security departments for digital surveillance, which facilitated industrial upgrading.

The paper proceeds as follows. We start by situating the question of how China was able to rapidly achieve industrial upgrading in the surveillance sector within the political economy of development literature. While traditional state-centric variables remain relevant, post-developmental state studies have delved more deeply into the issues of knowledge transfer, firm-level incentives, and local policy choices in a globalizing context. Building on these observations, we propose that both the timing of initial sectoral development and subnational economic conditions affect the process of “strategic adaptation” by local authorities and firms. Next, we explain how the national goal of constructing a robust surveillance apparatus contributed to the rise of a massive security market in China, albeit not through the conventional drivers of industrial policy. Based on original fieldwork conducted between 2017 and 2020, the empirical core of the paper compares development of the surveillance equipment industry in the provinces of Guangdong and Zhejiang. The analysis shows how political and economic conditions during different time periods created distinct industrial structures, including segmentation of the security market between private and public consumers. This market segmentation, in turn, created feedback effects that reinforced provincial variation in local government policies and business strategies.

## Explaining Industrial Upgrading and Indigenous Innovation

What do we know about what it takes for late developers to climb the global value chain? Over the past three decades, the literature has shifted from state-centric frameworks to the recognition that both government policies and firm strategies are critical for leveraging market opportunities, both domestically and globally. Yet local variation in regulatory and market conditions also mediates the ecosystem for industrial upgrading and firm-level strategies.

During the 1980s and 1990s, many political economists privileged the role of the state in industrial development by attributing the success of late industrializers to an independent and meritocratic bureaucracy, market-conforming state interventions, economic pilots, and effective regulatory institutions (Amsden 1992; Johnson 1982; Wade 1990; Woo-Cumings 1999). Some also emphasized the necessity of “embedded autonomy” or “governed interdependence” between state and society (Evans 1995; Weiss and Hobson 1995) in a delicate balance between incentives and discipline designed by capable and loyal bureaucrats (Ha and Kang 2011). In this statist stance, potential strategic responses by firms to upgrade their technological capabilities were undertheorized.

The popularity of classic developmental state explanations waned following the Asian financial crisis and the apparent globalization of neoliberalism (Haggard 2015). Various critiques and amendments of the developmental state framework ensued. For example, Joseph Wong’s (2011) “post-developmental state” study of the biotechnology sector found that bureaucrats are not adept at identifying which technologies should be supported to achieve successful innovation, resulting in waste of government resources and failed industrial policies.

By the time that China embarked on liberalizing reforms, Whittaker et al. (2020) observe that its economy had experienced “compressed development,” whereby manufacturers rapidly achieved “thin industrialization” by imitating foreign technology. Unlike the more restrictive FDI policies of its regional neighbors, China’s openness to investment by MNCs enabled domestic companies to participate in low-end segments of the GVC as product suppliers even faster than East Asia’s post-war industrializers (Chen 2018; Fu and Gong 2011; Lewin et al. 2016; Whittaker et al. 2020). Meanwhile, China’s enormous domestic middle-end market incentivized indigenous enterprises in certain sectors to climb the GVC by cooperating and competing with foreign companies (Brandt and Thun 2010). Through a process that Henry Wai-chung Yeung (2016) calls, “strategic coupling,” over time the most successful industrial sectors in East Asia have achieved industrial upgrading by partnering with leading foreign firms and embedding themselves in global production networks.

International market pressure can also spur fruitful partnerships between state and non-state actors domestically. Facing competition from abroad, new modes of public and private cooperation emerged in Korea and Taiwan’s green energy sector, creating “hybridized industrial ecosystems” that “link up all segments of the production and innovation value chain” (Kim 2019, 3; Kim and Thurbon 2015). Government-guided collaboration between research institutes and industry to commercialize cutting-edge technology is not unique to East Asia. Fred Block (2008) points to the synergy between US public institutions and private companies in facilitating commercialization coordinated by a “hidden developmental state.” The density of networks connecting the government with small- and medium-sized enterprises (SMEs), universities, and research institutions has blurred the boundaries between the public and private sectors, thereby obscuring the role of the government behind the scenes (Block 2008; Schrank and Whitford 2009). In her depiction of America’s “national security state,” Linda Weiss (2014) similarly observes that hybrid, public–private institutions strategically facilitated dual-use innovation in security-commercial technologies. Hybridity also obscures the visibility of public financing in security governance in the European Union’s drone industry (Martins and Küsters 2019).

While many scholars highlight the role of security politics in promoting innovation around the world (Taylor 2016), our study focuses on how the domestic security needs of an authoritarian regime contributed to the advancement of specific technologies. This raises the question of whether innovation is more likely in industries critical for national or domestic security. Superficial parallels exist between the US’s military-industrial complex and China’s domestic surveillance apparatus. Driven by geopolitical imperatives during the Cold War, American military expenditures also served to promote technological advancements in the defense industry through outsourcing contracts to SMEs (Block 2008; Cypher 1987; Weiss 2014). Following a cognate logic, large-scale public procurement of security equipment by China’s PSB incentivized certain domestic firms to invest strategically in developing state-of-the-art surveillance and crime prevention technologies. But the mechanisms operate at different levels of analysis.

A counterintuitive structural difference between the US and China is that even though the US has a federal political system, the nature of its national security state (Weiss 2014) rests on direct relationships between federal agencies and private contractors rather than mediated by state governments. Meanwhile, although China has a unitary political system, its government-business interactions occur in a more decentralized manner. Industrial upgrading depends on coordinating among vertical and fragmented bureaucracies and across localities with varying political economic conditions (Brandt and Rawski 2019; Breznitz and Murphree 2011; Rithmire 2014; Segal 2003; Thun 2006; Zhang and Peck 2015). When local bureaucrats possess the autonomy and administrative ability to implement policies tailored to local conditions, regionalism may facilitate industrial development (Pearson 2019; Segal and Thun 2001; Zhang 2020). However, local protectionism can hinder specialization, give rise to regional trade barriers, and compromise production based on comparative advantage (Bai et al. 2004; Young 2000). Corruption in the form of bureaucratic predation may also inhibit industrial development (Lin 2001).

Existing research suggests that the relative success of industrial upgrading in different localities depends on the nature of bureaucratic alliance strategies between government entities and various types of enterprises. When local governments ally with small foreign companies, Chen (2018) finds that is more likely to bridge the technology gap between global firms and local ones—and help domestic firms achieve industrial upgrading—than when localities choose to partner with world-leading MNCs (cf. Fuller 2016).

Yet the political economy of regional development is not a static phenomenon. Scholars have examined how actors (officials or firms) make strategic decisions, the consequences of those choices under different historical conditions, and whether policy decisions and corporate strategies conducive to upgrading or innovation are selected (Ang 2016; Chen 2018; Pearson 2019; Shen and Tsai 2016). Among these, an exemplary study is Yuen Yuen Ang’s (2016) “coevolutionary” approach to tracing the interaction between local state agents and market forces. Recognizing that these interactions are embedded in varying types of localities across different stages of China’s reform era provides a contextualized basis for understanding why local officials pursue certain developmental strategies, and how they adapt to changing market conditions.

In the spirit of this coevolutionary stance, this paper proposes a “strategic adaptation” approach to explain subnational variation in industrial upgrading. Our analytical framework comprises two main components. First, we focus on *government-business interactions driven by the domestic market*, unlike Yeung’s (2016) notion of “strategic coupling,” which emphasizes cooperation with foreign firms. Second, drawing on historical institutionalism and evolutionary approaches, we emphasize the role of timing and sequencing in explaining different developmental paths (Ang 2016; Pierson 2004; Mahoney and Thelen 2010; Whittaker et al. 2020). We contend that the initial phase of industrial development restricts the strategic options of local governments and enterprises. Local government policies enacted in response to these initial industrial conditions then mediate the business and innovation strategies taken by enterprises. Over time, variation in industrial development can be explained by the calculus of local actors as they pursue certain courses of action within the context of their local environments in a feedback loop. We conceptualize this decision-making process by subnational actors operating under specific spatial and temporal conditions as “strategic adaptation.” Unlike the term’s usage in the business management literature (e.g., Schindehutte and Morris 2001), we do not assume that adaptation leads to a normatively positive outcome. “Adaptation” by state and economic actors entails learning based on organizational experiences and responses tailored to shifts in the external environment. Chosen strategies may prove to be maladaptive, depending on other variables, but they are “strategic” in the sense of accounting for existing resources and perceived opportunities.

## Comparing China’s Economic Engines

The decentralized nature of China’s political economy has led to subnational variation and segmentation in the security industry, with divergent implications for technological innovation. To explore the mechanisms underlying this divergence, we selected two provinces and employ a most similar systems comparative research design (J.S. Mill’s “method of difference”). Guangdong and Zhejiang share attributes that should be conducive to fostering a technology-friendly ecosystem (Ma 2020; Zhang and Peck 2015). First, they are the most important manufacturing centers for China’s security industry and have local economies that are hospitable to both private- and foreign-invested enterprises. Second, both provinces have enacted policies that support high-tech enterprises, including preferential taxation, and subsidies from the science and technology department. Third, both provinces possess the educational and research infrastructure to support collaboration between industry and scientists. Fourth, both local governments have made major investments in their surveillance infrastructure.

Given these similarities, it is not surprising that both provinces have publicly listed security companies. Yet Guangdong and Zhejiang have developed different market structures, and their enterprises have taken divergent paths in their quest for industrial upgrading. During field interviews, business managers and public security officials observed, “Guangdong has a lot of small shrimp, but the dragon heads are in Zhejiang” (No. 33; No. 46). In other words, Guangdong has a large number of SMEs, while Zhejiang has fewer security companies, but they are the ones producing cutting-edge technology (Table 1). This outcome is less intuitive. Given Guangdong’s larger industrial base, first-mover development, and closer cooperation with foreign manufacturers, why were enterprises in Zhejiang able to surpass Guangdong in producing more competitive and technologically sophisticated security firms? Indeed, the logic of strategic coupling suggests that Guangdong’s earlier access to foreign technology and partners could have enabled their firms to be more competitive than those in Zhejiang in the surveillance equipment GVC. Our contrarian finding merits explanation.

**Table 1:** Registered security enterprises in Guangdong and Zhejiang (2003–2015)

Enterprise type	Province	2003	2004	2005	2007	2008	2010	2011	2012	2013	2014	2015
Number of security production enterprises	Guangdong	363	–	442	246	317	124	104	177	178	128	98
	Zhejiang	140	–	60	51	50	58	33	33	33	49	27
Number of security integration enterprises	Guangdong	230	523	1434	1528	1721	1543	962	2144	2437	1368	1438
	Zhejiang	290	416	448	315	655	693	769	828	1080	1108	1280

Source: *Chinese Security and Protection Industry Yearbook *2003–2005, 2007–2008, 2010–2015. Due to the cancellation of registration requirements, comparable data is not available after 2015.

To develop an empirically grounded understanding of this variation in industrial performance, from 2017 to 2020, we conducted field research in Guangzhou, Shenzhen, Dongguan, and Hangzhou—the cities where security enterprises are concentrated in Guangdong and Zhejiang, respectively. In addition to interviewing 91 local entrepreneurs, government officials, retired PSB cadres, and researchers, we consulted relevant government documents, industry yearbooks, corporate annual reports, and trade publications.^5^

## China’s Security State and Industrial Development

**Fig. 1:**
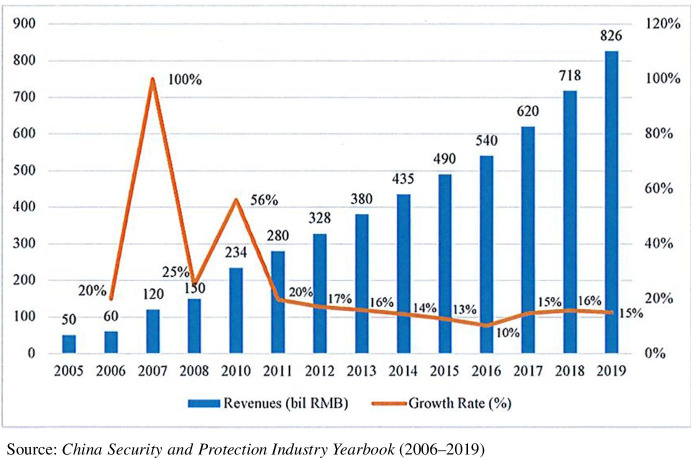
Revenue growth in China’s security industry, 2005–2019. Source: *China Security and Protection Industry Yearbook* (2006–2019)

Before discussing local variation, China’s security equipment industry should be understood within the context of the authoritarian regime’s broader stability maintenance and comprehensive governance agenda (Wang and Minzer 2015; Yang 2017). The party-state’s continuous investment in public security projects and construction of surveillance infrastructure have fueled an expanding market for security equipment and services. Government purchases account for nearly 60% of the revenues of China’s surveillance equipment firms (Millar 2015, 39). Despite declining GDP growth in recent years, Fig. 1 shows double-digit annual growth in the revenues of domestic security companies during 2005–2019.

Previous studies have attributed the failure of China’s high-tech industrial policy to bureaucrats’ weak understanding of technology, as well as the disincentivizing impact of local clientelism and protectionism for companies to engage in innovation (Segal 2003; Fuller 2016). In the security equipment sector, however, such problems have been largely averted due to the strong influence of experts in the PSB with advanced degrees in fields such as mechanical and electrical engineering, information science, wireless technology, and computer science.^6^ As frontline users of public security products, these technocrats evaluate the technical content, effectiveness, and stability of the product as the criteria for selecting “the winners”—regardless of the firm’s ownership status. As such, private enterprises that have generated the latest technological innovations have been invited to join the national and/or local standards committees to formulate product standards (No. 3; No. 18).

Some of China’s most capable technocrats work in research institutes under the Ministry of Public Security (MPS) and provincial PSBs that co-manage the central Security and Defense Standardization Technology (SDST) Committee. The latter includes the National Alarm System Standardization Technology Committee (SAC/TC100) and the Human Body Biologic Identification Standardization Technology Committee (SAC/TC100/S2). SAC/TC100 is responsible for setting security industry standards, including those for surveillance camera technology, city alarm systems, and monitoring network systems. By 2016, it had formulated over 120 industry standards.

Table 2 shows that *technocrats from the MPS and PSB* rather than cadres from the industrial planning bureaucracies (e.g., the Ministry of Industry and Information Technology or the National Development and Reform Commission) have maintained a steady and long-term role in the establishment of industry standards. After 2008, the participation of private entrepreneurs in setting standards increased markedly, and during 2014–2019, private and foreign enterprises accounted for 45% of the SAC/TC100’s representatives. By 2019, nearly all the affiliate members of this technology committee were from the private sector. Affiliates attend committee meetings ex officio and those demonstrating knowledge of the latest technologies may be selected into the committee for a 5-year term.

**Table 2:** Organizational structure of China’s SecurityStandardization Technology Committee

National Alarm System Standardization Technology Committee (SAC/TC100)				Human Body Biologic Identification Standardization Technology Committee (SAC/TC100/S2)	
Position^*^	*2002–2007*	*2008–2013*	*2014–2019*	*2007–2012*	*2012–2017*
Cadre	8	13	15	–	–
Technocrat	23	22	25	11	–
Expert	5	6	7	10	–
SOE manager	2	4	3	2	–
MOE manager	2	4	5	–	–
Private entrepreneur	12	38	42	7	–
FE manager	–	2	3	–	–
Total	52	89	100	30	42

*Cadres include government officials without any title of technical post. *Technocrats* refer to government officials with title of engineer or researcher. *Experts* include professors or researchers in university/research institutions. *SOE managers* hold managerial positions in state-owned enterprises. *MOE manager* refers to managers of mixed ownership enterprises. *Private entrepreneurs* include general managers or general engineers of private enterprises. *FE managers* work in foreign enterprises or Chinese-foreign joint ventures. Sources: Official documents of National Alarm System Standardization Technology Committee (STC) (SAC/TC100) and Human Body Biologic Identification STC (SAC/TC100/S2)

Hard constraints on technology have made China’s procurement of public security products a more competitive process than one might expect given the sensitivity of their functions. During the early years of public security infrastructure construction (2005–2008), the SAC/TC100 issued a procurement list recommending domestic video surveillance companies. This practice was subsequently abandoned when provincial PSBs objected that limiting eligible suppliers to local firms might prevent them from purchasing equipment from more competitive sources. In general, at least through 2010, if the products of specific companies met the government’s industry standards, then they were included on the approved procurement list of state-owned enterprises (SOEs) and/or government agencies. For public projects, all product manufacturers meeting the standard may compete in the tender process (No. 9; No. 33).

Once the MPS defined certain technical standards, they became required for PSBs throughout the country, thereby creating a uniform basis for procuring public security products. The existence of national industrial standards has been helpful for security equipment producers, especially those focusing on design and production. Although local protectionism exists in security integration and engineering,^7^ it has not eliminated the space for industrial development and innovation. China’s security market is dominated by a small number of equipment designers and producers that can formulate national industrial rules and standards.

Starting in the late 1990s, the central government decentralized approval of security equipment production and sales to provincial PSBs. Entrepreneurs seeking to establish a security equipment company would contact the municipal PSB for preliminary review and await approval from the provincial PSB. To engage in equipment production legally, a company needs one of the following three administrative licenses: (1) a production license issued by the national or a local technical supervision department; (2) a China Compulsory Certificate (CCC) mark issued by the MPS or national certification center; or (3) a production approval certificate issued by the local public security department. Nominally, the public security and technical supervision departments are authorized to examine and approve security products. But in practice, many SMEs engaged in assembling entry-level security products never applied for PSB approval. They simply started operating after registering their businesses with the local Industrial and Commercial Management Bureau (No. 6; No. 8). An equipment manufacturer explained, “When we first started our business in the early 2000s, we knew it would be too difficult to get certified to produce products for the public security market. Instead, we only registered for a regular business license (No. 9).” This practice was echoed by other interviewees, especially in Shenzhen where security equipment producers were active in reverse engineering foreign electronics (No. 10; No. 32; No. 61).

## Earlier, Not Better: Strategic Adaptation in Guangdong

When the Chinese government turned to technology to build a more robust surveillance infrastructure after 1989, Guangdong was the first province to develop a security industry due to its locational advantages and earlier start on economic reform. Initially, Guangdong’s security companies mainly engaged in leasing or selling overseas products. In the process, some companies learned the foreign technology for original equipment manufacturing (OEM) production, meaning the capacity to manufacture components of foreign security products. Economic development and the influx of rural migrant workers to Guangdong also enhanced demand by local governments and the private sector for digital surveillance and security construction. This created two types of local markets, serving public versus private security needs, respectively. The PSB’s installation of public surveillance units augmented the coercive capacity of local governments that were struggling with a low per capita ratio of police (Greitens 2017). Meanwhile, the private segment of the security market served retail stores/malls, commercial offices, and residential buildings.

From the late 1990s to mid-2000s, two security industry markets led by Guangzhou and Shenzhen gradually formed in Guangdong. Due to a large concentration of engineering firms in Guangzhou, private producers with official backgrounds took the lead in meeting the demand for equipment by government units. Those types of entrepreneurs tended to be low-level cadres before setting up their own companies. Others were former rank-and-file managers and workers of state factories who entered the private sector due to the dissolution of SOEs and administrative agencies. Lacking wage employment, they forced themselves to redeploy their skills to build up businesses. A former front-line director of a loudspeaker factory recounted, “I was only 30 years old when I was laid-off and needed to do something else. Because I have some knowledge of electrical work, I borrowed money from relatives in my hometown [Jiangmen] and moved to Guangzhou to sell security alarms used in buildings (No. 13).” Others learned first-hand information from working in the public sector and identified business opportunities in the security industry (No. 14). An ex-PSB cadre shared, “I knew that public security departments have a tremendous need for surveillance products, so I started a business with a Taiwanese partner to produce video surveillance cameras (No. 19).”

Meanwhile, Shenzhen had a concentration of medium-sized OEMs, small-scale assembly companies, distributors, and exhibitors that were oriented towards meeting the demand for surveillance products in the civilian, commercial security market. Many enterprises in Shenzhen were clustered in areas where the manufacturing chain for technology equipment assembly production was well developed and hospitable for start-up businesses. Such indigenous entrepreneurs entered this field due to the low threshold for producing civilian security products such as alarms, electronic locks, and safes. Several enterprises grew out of earlier workshops that specialized in copying and replicating foreign products. Others launched their businesses by leasing foreign security equipment. It was not until after the 2010s—when the Chinese government encouraged using domestic security products—that Guangdong’s producers started investing in developing their own brands of equipment (No. 9; No. 12).

### Comfortable Market Position, Short-Sighted Corporate Strategies

Guangdong’s vast and segmented security market allowed for a diversity of SMEs to coexist, including OEMs, foreign product agents, and engineering contractors. The large market meant that manufacturers in Guangdong did not feel pressured to upgrade their research and development (R&D) capabilities. Cooperation between local and overseas companies in Guangdong took the form of product distribution and OEM. Because local products did not meet the PSB’s technical requirements, early public security projects in the Pearl River Delta relied on imports. Both provincial and local officials had more confidence in the quality of foreign products at that time. “Frankly,” a PSB technocrat told us, “We did not even consider local suppliers because we knew that Samsung and Sony had higher quality and more advanced products (No. 27).” Until the mid-2000s, local companies still found it lucrative to distribute or simply copy foreign surveillance products. The relative ease and profitability of this method diminished the motivation of OEM producers and product agencies to develop their own technology (No. 3; No. 23).

Furthermore, rapid and on-going construction of commercial real estate in the Pearl River Delta provided a vigorous source of demand for Guangdong’s civilian market in security equipment manufacturing. Most security factories developed products for building automation, parking access, office access, shopping mall security, and so on. Due to the relatively low technical requirements of the civilian market, most producers focused on reducing the cost of materials, designing the products’ external appearance, and enhancing private users’ experience rather than incorporating cutting-edge technology. The few companies engaged in R&D of electronic surveillance cameras were similarly attracted to the booming retail market and did not devote resources to improving the technical qualities that PSBs valued such as video definition, hard drive capacity, sensor sensitivity, etc. (No. 48). The huge demand within Guangdong itself made most local enterprises complacent with the developmental status quo. As a result, from the early to mid-2000s, local factories maintained small production lines with a limited range of products (No. 11; No. 20).

The market strategy of Guangdong’s enterprises to serve commercial clients is reflected in the distribution of patents. The major security equipment manufacturers in Guangdong, such as Anjubao, Sunell, and Wanjiaan, have a very high proportion of lower end (*design* and *utility model*) patents, while their share of *invention* patents with higher technical requirements is less than 50% (Table 3).

**Table 3:** Types of patents owned by major security firms, 2019

	Invention	Utility model	Design	Location
Hikvision	1504	871	1294	Zhejiang
Uniview	1282	184	176	Zhejiang
Dahua	1199	548	491	Zhejiang
Gosuncn	256	95	32	Guangdong
Infinova	201	59	29	Guangdong
Pcitech	189	0	4	Guangdong
Haoyun	98	48	15	Guangdong
Anjubao	59	90	158	Guangdong
GQY	56	15	9	Zhejiang
Joyware	47	31	21	Zhejiang
Dali	35	16	12	Zhejiang
Sunell	22	44	68	Guangdong
Wanjiaan	21	16	27	Guangdong

*Invention* patents represent the most original innovations to a technology or product. *Utility model* patents are issued for minor improvements or adaptations to existing products. *Design patents* refer to the external appearance of the product.

Source: National Intellectual Property Administration: http://www.sipo.gov.cn/; patents database from Biaten: https://www.baiten.cn/

### Disincentivizing Effects of Regulation on Innovation

Before the mid-2000s, over half of China’s security companies were based in Guangdong. Provincial PSB officials that we interviewed indicated that the early stage of the security industry was “very chaotic” (*hunluan*) with rampant infringement of intellectual property rights (IPR), unlicensed production, and unauthorized security installations (No. 4; No. 7). SMEs admitted that their early products were largely derived from copying similar ones from overseas. When local enterprises introduced their own products, they became embroiled in IPR disputes with foreign partners. Heated accusations and even litigation also occurred among local businesses (No. 10; No. 21). To reduce commercial disputes and ensure proper registration of manufacturers, in 2002, the Guangdong People’s Congress issued the country’s first and only provincial security industry regulatory law entitled, “Guangdong Province Regulations for Security Technology and Defense Management.” Unlike other provinces that had minimal administrative regulations within their local PSBs, the formal status of Guangdong’s law enabled its PSB to manage the province’s security industry in a more orderly manner.

However, the introduction of these provincial regulations inadvertently curbed the development of its security companies into more technologically sophisticated niches of the market. Local PSBs ended up adopting a conservative attitude towards the introduction of new security products to reduce their regulatory responsibilities, which includes issuing production licenses to enterprises.^8^ Compounding this bureaucratic reluctance, Guangdong’s application procedure for proposing corporate standards is extremely cumbersome, involving multiple rounds of review and certification. Security equipment producers adapted strategically to these new policy constraints. Rather than proposing their own technical standards and developing new products, it was a better use of time and resources to avoid red tape and manufacture pre-existing prototypes. A private entrepreneur complained,

I once spent half a year trying to get product certification, running back and forth between various departments. The cost for me was too high. I am a person who abides by the rules. I will not put products on the market like others without obtaining official documents. But this proved to be very detrimental to my business development (No. 47).

Guangdong’s innovation-dampening regulatory environment contrasts sharply from the one that evolved in Zhejiang nearly one decade later.

## Latecomer Advantage: Strategic Adaption in Zhejiang

Unlike the initial phase of Guangdong’s security industry when a diversity of companies flourished at multiple segments of the production and distribution chain, small manufacturers dominated Zhejiang’s industry from the outset. They were concentrated in the provincial capital of Hangzhou, a city that benefited from Mao-era state investment. This legacy provided a favorable research infrastructure for technological innovation, especially after privatization of SOEs and collective enterprises in the early 2000s. Indeed, the founders of China’s largest surveillance manufacturers, Hikvision and Dahua, have state-sector lineages. Before becoming the general manager of Hikvision, Hu Yangzhong was a researcher at the No. 52 Institute of the Chinese Electronics Information Company. The founder of Dahua was a technical cadre in a local state-owned electronics equipment factory. When he set up his own video surveillance company, he recruited its research team from the SOE. Coupled with the density of technical talent in Hangzhou, the loosely regulated market environment ultimately provided a more favorable ecosystem for upgrading in the security industry.^9^

### Relaxed Regulation of a Competitive Market

Since the number of security companies in Zhejiang was relatively small, competition was not intense during the early stages of development. As latecomers, security equipment manufacturers in Zhejiang shared a sense of local solidarity in needing to catch up and cooperated in setting both industry-wide and local standards (No. 59). A company engineer recalled, “In the early days, everyone was a small company. Although there were overlapping areas of competition, it was difficult for everyone to develop well if there was no breakthrough in technical standards (No. 43).”

Contrary to developmental state expectations, the government of Zhejiang did not pay attention to the security equipment industry until it had already taken off. In what is best described as benign neglect, provincial authorities did not impede the development of new products and standards. An entrepreneur admitted,

The cluster development of Hangzhou’s security companies emerged inadvertently. Companies such as Hikvision, Dahua, Ebi, Dali, Nanwang, and Red Apple emerged and scaled up without much notice by the government (Gao and Hu 2008, 38).

Unlike security companies in Guangdong, those in Zhejiang are permitted to have their proposed standards peer-reviewed by experts selected by themselves without extra formalities, which has motivated local manufacturers to improve their product standards (No. 51). By 2015, enterprises in Zhejiang were authorized to produce 1432 different types of security equipment, 86% of which are produced by two domestic giants, Hikvision and Dahua. That same year, only 373 categories of equipment were officially licensed in Guangdong, and two of the major license holders, Honeywell and Infinova, were American-invested joint ventures. Lax regulatory conditions allowed Zhejiang enterprises to accelerate iteration of their products and identify emerging market opportunities. A market analyst explained, “In Zhejiang, companies can quickly obtain product certifications and launch them on the market. Even if the products are not good enough, they can quickly adjust the follow-up strategy (No.52).” In Guangdong, by contrast, potential new products often missed the right time to enter the market due to the lengthy approval process for official certifications.

### Innovating for the Government Procurement Market

In addition to differences in their regulatory environments, the manner in which Zhejiang’s security industry gradually surpassed Guangdong’s can be traced to variation in local corporate strategies. This is evident by developments in the video surveillance sector. While Guangdong’s earlier firms mainly produced infrared video cameras during the mid-2000s, Hikvision and Dahua recognized the demand for digital video recorder (DVR) technology in the construction of public video surveillance. Both companies thus invested heavily in DVR-related R&D.

When Hikvision and Dahua were newly established in 2001, enterprises based in Guangdong (including foreign-invested ones) accounted for nearly 70% of the national surveillance equipment market. At the time, major public areas in Zhejiang used surveillance cameras produced by another local company, Nanwang, which is now bankrupt. To increase their market share of public surveillance equipment, the two companies scrutinized blueprints for constructing public video surveillance platforms and made technical improvements and innovations accordingly. Dahua’s CEO, Fu Liquan, reflected on the company’s perspective,

During the war era, one could contribute to the country by joining the army. During peaceful times, enterprises can serve the country through industry. Dahua can contribute to national projects and provide stable and reliable digital video equipment, which is a means for our industry to serve the country (Fu and Guo 2008, 14).

He found inspiration from defects in card-type DVRs commonly used in prisons and state-owned banks: “If public institutions such as banks and prisons can use our alternative products, it will be a huge market with great prospects” (Fu and Guo 2008, 10). Following this due diligence, in 2002, Dahua launched an embedded 8-channel DVR with more stable storage of images and released a 16-channel DVR one year later.

Concurrently, Hikvision initiated R&D on video compression technology. When the MPEG-4 compression format for low-bandwidth recording was mainstream in the market, Hikvision introduced the world’s first H.264 video compression standard and combined it with digital pre-distortion settings (DPS). This innovation further improved the definition of surveillance video while occupying less network bandwidth (Hu and Lei 2011). As a result, Hikvision won multiple government orders in the Safe Cities national initiative launched in 2005.

The early innovations of Hikvision and Dahua did not involve many original technologies, as they merely recombined surveillance products with existing technologies not used by other overseas manufacturers. Because foreign suppliers were not attuned to local information, they believed that their level of video surveillance technology was sufficient for public sector use and did not perceive an urgent need to upgrade video compression and data transmission technology. The sluggishness of foreign companies led them to lose contracts to the two new companies in Zhejiang.

Our research indicates that Dahua and Hikvision’s success in the public security procurement market was not due to techno-nationalist industrial policy that privileged domestic producers. In fact, during the initial phase of Safe City construction, the Hangzhou PSB used surveillance systems from H3C, a joint venture between Huawei’s security unit and the US company 3Com. Furthermore, even though cities in Guangdong had been loyal consumers of foreign security equipment until the 2000s, their city governments were the first to use Dahua and Hikvision products outside Zhejiang. Tracing their success in entering Guangdong’s public surveillance construction market reveals the mechanisms through which the two Hangzhou companies expanded into the national market.

Local PSBs in Guangdong tended to fulfill the purchasing targets of security products based on quality rather than the source of the product. A seasoned PSB technocrat who has evaluated multiple government monitoring projects insisted, “We decide how to award a contract by assessing *only* the technical parameters and durability of the product. Whether the product is produced by local companies does not factor into our evaluation standards (No. 29).” His defensive tone reflected frustration with earlier incidents of local protectionism. During the mid-2000s, the Guangzhou Science and Information Bureau issued a list prioritizing procurement from local companies for public video surveillance. When the city-level PSBs found that these local companies could not meet the requirements for monitoring public security, they asked the provincial PSB to instruct the local Science and Technology Department to cease their promotion efforts. A technocrat responsible for the construction standards of Guangdong’s public security video surveillance explained, “At that time, first Dahua, and then Hikvision, were the only ones with the technology to provide 16-channel DVR with up to 26 frames in each display. Other overseas products could not deliver such images. That is why the PSBs chose their products (No. 48).”

Once China’s national surveillance Skynet Project was fully rolled out during the 2010s, security firms in Zhejiang benefited the most. Guangdong’s companies were not devoid of capacity to cater to public security needs. Instead, they made strategic choices appropriate for the vast consumer market. For example, the Jinpeng Security Company in Guangzhou already produced DVR in the early 2000s. Instead of concentrating its R&D on surveillance technology, however, it chose to develop civilian communications products concurrently (No. 14). Jinpeng then fell behind its competitors in Zhejiang technologically and missed a massive window of opportunity during the mid-2000s. Two other companies, Raysharp and TVT, gradually became original design manufacturers (ODMs) in the production of customized security devices for overseas customers. Although the expansion of surveillance construction projects helped to transform some of Guangdong’s security integrators (e.g., Gosuncn and Pcitech) into high-tech companies offering internet-of-things (IoT) and video analysis technologies, these integrators depend entirely on upstream hardware and software vendors.^10^ Table 4 shows the huge gap between Guangdong and Zhejiang in industrial innovation capabilities. The total number of patents of all major enterprises in Guangdong significantly lags that of Dahua, which ranks third in Zhejiang. Moreover, Guangdong’s enterprises are more dependent on acquiring patents rather than generating independent R&D.

**Table 4:** Independent patents and acquired patents of major companies

Name	Independent patents	Acquisitions	Total	PTC*
**Zhejiang**				
Hikvision	4827	113	4940	315
Uniview	2758	23	2781	
Dahua	1707	150	1857	15
Joyware	101	7	108	2
GQV	81		81	
Dali	64	2	66	
Zeno	12	15	27	
**Total**	**9550**	**310**	**9860**	**332**
**Guangdong**				
Gosuncn	310	98	408	3
Anjubao	307	10	317	2
Infinova	290	5	295	
Pcitech	189	9	198	1
Haoyun	100	66	166	
Sunell	127	14	141	
TVT	126		126	
Raysharp	82	28	110	
Wanjiaan	37	32	69	
Aebell	15	24	39	
**Total**	**1583**	**286**	**1869**	**6**

*PTC refers to the Patent Cooperation Treaty, which protects patents in all 150 Contracting States simultaneously (see the World Intellectual Property Organization’s explanation at: https://www.wipo.int/pct/en/faqs/faqs.html). Sources: National Intellectual Property Administration: http://www.sipo.gov.cn/; patents database from Biaten: https://www.baiten.cn/

Determined to ensure public security and political stability, the Chinese government drove the need for surveillance and security equipment with advanced technology to deliver image clarity, identification sensitivity, structured video analysis, crowd and facial recognition, and high-speed transmission. In the process, public security surveillance projects were extended from major cities to counties and even villages. Convergence between the government’s security agenda and corporate innovation strategy accelerated the capacity of Zhejiang’s security companies to achieve faster intergenerational updates than those in other countries. Especially in the transformation from analog to digital technology, Zhejiang’s security giants took the lead in upgrading from uncompressed digital camera technology to compressed video network technology that is better suited for constructing Smart Cities than that offered by international competitors. As urban surveillance expanded throughout the world, Zhejiang’s proprietary brands developed a competitive advantage in the GVC due to their accumulated technological capabilities in adapting to the Chinese government’s surveillance requirements (Table 5). Most companies in Guangdong, by contrast, only served domestic customers, with the exception of ODM firms that export their products.

**Table 5:** Domestic versus foreign revenues of listed security companies, 2016 (unit: million RMB)

Name	Domestic revenue	Ratio	Foreign revenue	Ratio
**Zhejiang**				
Hikvision	22,563	71%	9360	29%
Dahua	8273	62%	5055	38%
Uniview	1768	86%	292	14%
Dali	315	93%	23	7%
Joyware	295	100%	–	–
GQY	172	100%	–	–
**Guangdong**				
Pcitech	2840	100%	–	–
Gosuncn	1307	100%	–	–
Anjubao	791	99%	7	1%
Haoyun	544	100%	–	–
Infinova	488	25%	1490	75%
Raysharp	251	27%	680	73%
TVT	37	7%	488	93%

Source: Annual reports of various companies.

## Explaining Divergent Provincial Paths

The divergent paths of industrial development in Guangdong and Zhejiang demonstrate the relevance of provincial-level conditions in a manner that produced self-reinforcing feedback effects (Table 6). Although Guangdong seemed to possess advantageous conditions for industrial upgrading, it has yet to produce globally leading companies, while Zhejiang’s younger enterprises climbed to the top of the GVC. Timing played a counterintuitive role in this process. Because Guangdong developed earlier, technocrats chose tougher regulatory policies to tame a large and messy free-wheeling market. These provincial regulations unintentionally disincentivized product innovation and diversification by local enterprises. Given Guangdong’s more developed market of private sector consumers, its enterprises opted to develop technology catering to the civilian market rather than the public sector. While this strategy made sense in the context of restrictive provincial policies, local enterprises lost opportunities to obtain government outsourcing contracts.

**Table 6:** Comparison of local strategic adaptation between Guangdong and Zhejiang’s security industry

Locality	Initial industrial condition	Firm strategies	Local bureaucrat choices	Firm strategies	Industrial upgrading outcome
Guangdong	Large number of OEMs, foreign product agents, engineering contractors, and small workshop producers	Fierce competition in a civilian consumer-oriented market and in integration	PSBs and other departments adopted strict approach in regulation and industrial management	Delayed response, whichhindered corporate R&D strategies for the public security market	Existence of a leading domestic IoT and surveillance industry, but not globally competitive
Zhejiang	Small number of domestic equipment producers established by engineers; friendly competition in local market	Corporate strategies mainly focused on public security market	PSBs and other departments were lax in regulation and industrial management	Strong motivation in R&D for new products and expanded business in public security market	Generated significant service and product providers in the global IoT and surveillance industry supply chain

In Zhejiang, where the industry developed later, the government adopted a more relaxed policy stance that encouraged local enterprises to be innovative and entrepreneurial in serving a public market. Regulatory laxity, rather than local state activism, facilitated upgrading. As such, the technological achievements of Zhejiang’s surveillance industry should not be attributed reflexively to developmental state industrial policy. *The local state did not select surveillance technology as a priority sector at the outset*. By the time Hangzhou built an impressive IoT Industrial Park in 2010 that welcomed local surveillance companies, Hikvision and Dahua firms already ranked among the top security firms globally. Favorable local state industrial policies followed, rather than preceded, the rise of China’s most competitive security firms. The coevolutionary sequence underlying Zhejiang’s path to technological innovation in video surveillance started with the combination of being a latecomer, the PSB’s demand for increasingly sophisticated products, and liberal provincial regulation. Taken together, these were the *local* contextual conditions that incentivized firm-level strategic adaptation to serve the largest public consumer of public surveillance, the Chinese government.

There was nothing inevitable or pre-determined about Zhejiang’s success in producing globally competitive security firms. During the 2000s, China’s urban surveillance projects were still dominated by foreign MNCs such as Samsung, Sony, Panasonic, Axis, and Honeywell. As Zhejiang’s security firms were initially emerging, PSBs preferred well-established international brands to support their mandate of completing Safe City construction within 3 to 5 years. Although corruption existed in outsourcing engineering contracts downstream in the industrial chain (Wang 2014), technocrats reviewing projects prioritized the robustness and usability of monitoring systems, which inhibited rent-seeking from harming product innovation at the front end of the chain. It is primarily within the past decade that escalation of geopolitical concerns triggered nationalistic procurement of products with sensitive technology. Following Edward Snowden’s revelation of US cyber espionage activities in 2013, China shifted more decisively towards purchasing domestically manufactured security equipment. By then, Zhejiang already had companies capable of meeting the technology requirements of China’s expanding surveillance party-state. The national security merits of domestic procurement aligned with capacity for indigenous innovation in a high-priority sector.

## Conclusion

The stunning rise of China’s security sector in the age of globalized production provides an opportunity to revisit the relationship between the state and industrial development in an era when the most value is generated by technology companies operating at the apex of global value chains. The fraternal twin concepts of the developmental state and state capitalism provide familiar, but insufficiently specified explanations for bridging the gap between centrally defined technological objectives and their realization by domestic firms. More recent concepts of “compressed development” and “strategic coupling” provide insight into how firms in contemporary late industrializers may enter different segments of the GVC. Security producers in Guangdong achieved “thin industrialization” early on by imitating foreign technology, and their dominance of China’s commercial market could plausibly explain why newcomer firms in another province sought out a different niche of the security market. Zhejiang’s surveillance giants benefited more from the latecomer advantages of compressed development, but our research indicates that they did not come to lead global rankings through strategic partnerships with multinationals. Instead, they recognized an opportunity to improve on existing foreign technology and had the local regulatory space to engage in technological innovation.

Our study demonstrates that while both national priorities and global competition create pressure for industrial upgrading, examining local bureaucratic preferences and firm-level adaptive strategies is equally relevant for understanding the process through which breakthrough technologies are incentivized and adopted—or not. For comparative purposes, identifying key variables in this coevolutionary process of sectoral development requires contextual specification. Given that mass government procurement correlated with technological breakthroughs in China’s surveillance industry, deriving developmental lessons from this case needs to be situated accordingly. Specifically, what are the conditions under which public procurement in the age of globalization may lead to success in industrial upgrading and technological innovation? The foregoing subnational comparison indicates two key variables: (1) the existence of a *specialized bureaucracy* empowered with the authority and staffed with the expertise to ensure high technical standards and (2) a *regulatory environment that is sufficiently permissive* to allow for rapid experimentation. Domestic firms facing this combination of conditions are better positioned and motivated to undertake innovation at the technological frontier of their particular sectors.

While these variables echo elements of classic developmental state logic, a substantive difference is that China’s industrial policy never targeted the surveillance sector as security manufacturers were proliferating in the late 1990s and ascending the GVC through the late 2000s. Instead, the proximate impetus for upgrading was steered by public security technocrats that were charged with mass construction of public surveillance, and empowered to insist that public procurement prioritize quality regardless of whether the manufacturers were foreign or domestic. Meanwhile, latecomer firms faced few local bureaucratic obstacles in introducing products that met the high technical standards of the PSB.^11^ Assessing the extent to which our argument is applicable to other frontier sectors thus involves a combination of public procurement, technocratic authority, foreign competition, and regulatory permissiveness.

Beyond the surveillance industry, dramatic expansion in the concept of “holistic national security” under Xi Jinping’s leadership provides ample scope for identifying potentially comparable sectors within China.^12^ Following a similar dynamic, public procurement of clean energy technology to promote China’s “resource security”^13^ has stimulated R&D and incentivized industrial upgrading among domestic firms in wind and solar power (Lewis 2013; Brandt and Wang 2019; Pearson 2019). Given the existence of several regional industrial clusters in the clean energy GVC (e.g., solar cells, wind turbines, and permanent magnets), our strategic adaptation framework can be applied to analyze variation in industrial upgrading across different provinces, including Guangdong, Fujian, Jiangxi, Inner Mongolia, Jiangsu, and Zhejiang.

Relatedly, in the interest of “ecological security,” the party-state has deployed increasingly coercive means to monitor industrial production and carbon emissions, while binding local governments to enforce pollution control targets (Li and Shapiro 2020). These trends have fueled procurement of IoT and digital surveillance technologies by the Environmental Protection Bureau (EPB, No. 71; No. 75), as well as mass procurement of pollution control and emissions reduction technologies by local governments. As local environmental protection departments grow in administrative authority and become the key gatekeeper of pollution prevention and control technologies, EPB technocrats have the potential to play a role akin to that of public security departments in incentivizing technological innovation among domestic firms. Indeed, our fieldwork in Guangdong indicates that the coercive authority of EPB offices has already increased markedly over the past decade (No. 70).

Ultimately, upgrading in the era of compressed development is an on-going ambition for economies exposed to global competition. We believe that focusing on the adaptive capacity and strategies of local governments and firms offers a finer-tuned level of analysis than national initiatives. For example, India’s Smart Cities project has encountered multiple obstacles due to the weak administrative authority of municipal governments, insufficient technical expertise, bureaucratic resistance, and shortage of qualified domestic suppliers (Fromhold-Eisebith and Eisebith 2019). By contrast, during the early 2010s, the regional government of Lombardy in Italy successfully experimented with using public procurement to incentivize technological innovation tailored towards applications in health care, energy, and agriculture (Vecchiato and Roveda 2014). Seeking higher value-added markets, firms in Lombardy participated in the process of identifying and developing new technologies. An OECD (2017) report on “Public Procurement for Innovation” similarly traces “good practice” examples of upgrading to specific subnational localities, including telemedicine in the region of North-Rhine Westphalia (Germany), hybrid lighting in the city of Jaroslaw (Poland), and smart personal protective systems in the Észak-Alföld region (Hungary). These positive cases of upgrading share the technocratic, regulatory, and strategic attributes identified in this study.

Globally, an increase in state-supported upgrading initiatives may be expected as popular backlash against globalization and China’s economic rise threaten to disrupt segments of GVCs with sensitive technology (Liu 2021). This can be seen in the banning of Huawei’s 5G wireless technology by the US and several other countries, and the race to develop 6G. Since 5G standards are leading edge and challenging to commercialize, however, most countries still procure from Huawei and ZTE while pressure to develop substitutes continues. The framework of local strategic adaptation in our study points to contemporary opportunities for upgrading as various supply chains are being renegotiated. Latecomer firms and regions are not necessarily disadvantaged if technocrats are able to overcome political tendencies towards local protectionism and regulatory red tape. During the recent development of covid-19 vaccines, a sense of urgency combined with the need for efficacy enabled relatively young pharmaceutical companies, BioNTech and Moderna, to introduce mRNA technology for mass vaccinations. Other challenges subject to local strategic adaptation lie ahead.

## Footnotes

1 With 50 million CCTV cameras installed, the USA has the highest density per capita at 15.28 cameras per 100 people. China has 14.36 cameras per 100 people (Baltrusaitis 2019).

2 The seven companies include Hikvision (ranked #1), Dahua (#2), Uniview (#6), Tiandy (#7), Infinova (#12), Zkteco (#14), and Streamax (#15).

3 By 2014, the market shares of Hikvision and Dahua in global security equipment were 9.0% and 3.3% respectively, ranking first and third in the world (Millar 2015).

4 Three Zhejiang-based companies—Hikvision, Dahua, and Uniview—account for 30% of global revenues in video surveillance (Alalouff 2018).

5 To protect the identity of research informants, direct quotations and references to information that they shared are indicated by a numerical code.

6 The older group (university graduates before 1996) were directly assigned to the corresponding posts by the government, who belonged to the generation known as “red engineers” (Andreas 2009). The younger group grew up during the reform era and entered relevant units through direct recruitment by the police department instead of the national civil servant examination.

7 Most entrepreneurs mentioned that competitive tenders of public agencies’ outsourcing projects are often more beneficial for local security companies that have strong ties with contract-issuing officials.

8 Although the local PSB issues production licenses for security equipment, they lack authority to sanction enterprises that produce illegally because enforcement authority resides with the Industrial and Commercial Management Bureau (No. 26).

9 During the early years, Zhejiang had easier access to the only two research institutes of MPS that were authorized with certifying security products in Beijing and Shanghai. Before transportation infrastructure was better developed, it took Guangdong enterprises up to 6 months to complete the approval process. Companies in Hangzhou, by contrast, could travel to Shanghai quite readily (No. 65; No. 77).

10 Because integrators are involved in the use and analysis of public data, they usually only serve their own national governments. The characteristics of integrators make it difficult for them to expand overseas markets like product manufacturers.

11 A recent empirical study similarly finds that surge in Chinese AI patents is highly correlated with public procurement of surveillance software (Beraja et al. 2020).

12 Introduced in 2014 and expanded afterward, Xi’s “holistic national security” includes 16 types of security, including technological security, ecological security, space security, cybersecurity, resource security, and biosecurity (cf. Wuthnow 2017).

13 Xi’s energy security strategy entails “the revolution of energy consumption, energy supply, energy technology, energy system, and international cooperation.” Increasing the supply and consumption of clean energy is a key part of this strategy (see: http://www.gov.cn/xinwen/2020-12/31/content_5575657.htm).
